# Production of Multiple Transgenic Yucatan Miniature Pigs Expressing Human Complement Regulatory Factors, Human CD55, CD59, and H-Transferase Genes

**DOI:** 10.1371/journal.pone.0063241

**Published:** 2013-05-21

**Authors:** Young-Hee Jeong, Chi-Hun Park, Gun-Hyuk Jang, Yeun-Ik Jeong, In-Sung Hwang, Yeon-woo Jeong, Yu-Kyung Kim, Taeyoung Shin, Nam-Hyung Kim, Sang-Hwan Hyun, Eui-Bae Jeung, Woo-Suk Hwang

**Affiliations:** 1 Sooam Biotech Research Foundation, Seoul, Republic of Korea; 2 Department of Animal Sciences, Chungbuk National University, Cheongju, Republic of Korea; 3 College of Veterinary Medicine, Chungbuk National University, Cheongju, Republic of Korea; University of Connecticut, United States of America

## Abstract

The present study was conducted to generate transgenic pigs coexpressing human CD55, CD59, and H-transferase (HT) using an IRES-mediated polycistronic vector. The study focused on hyperacute rejection (HAR) when considering clinical xenotransplantation as an alternative source for human organ transplants. In total, 35 transgenic cloned piglets were produced by somatic cell nuclear transfer (SCNT) and were confirmed for genomic integration of the transgenes from umbilical cord samples by PCR analysis. Eighteen swine umbilical vein endothelial cells (SUVEC) were isolated from umbilical cord veins freshly obtained from the piglets. We observed a higher expression of transgenes in the transgenic SUVEC (Tg SUVEC) compared with the human umbilical vein endothelial cells (HUVEC). Among these genes, HT and hCD59 were expressed at a higher level in the tested Tg organs compared with non-Tg control organs, but there was no difference in hCD55 expression between them. The transgenes in various organs of the Tg clones revealed organ-specific and spatial expression patterns. Using from 0 to 50% human serum solutions, we performed human complement-mediated cytolysis assays. The results showed that, overall, the Tg SUVEC tested had greater survival rates than did the non-Tg SUVEC, and the Tg SUVEC with higher HT expression levels tended to have more down-regulated α-Gal epitope expression, resulting in greater protection against cytotoxicity. By contrast, several Tg SUVEC with low CD55 expression exhibited a decreased resistance response to cytolysis. These results indicated that the levels of HT expression were inversely correlated with the levels of α-Gal epitope expression and that the combined expression of hCD55, hCD59, and HT proteins in SUVECs markedly enhances a protective response to human serum-mediated cytolysis. Taken together, these results suggest that combining a polycistronic vector system with SCNT methods provides a fast and efficient alternative for the generation of transgenic large animals with multiple genetic modifications.

## Introduction

Pig-to-human organ transplantation has arisen as a result of human organ donor shortages for transplantation. Despite substantial progress in the last two decades, the immune rejection processes underlying xenograft transplantation are not fully understood. Up to now, genetic modifications of several identified immune-related genes have been shown to reduce severe xenograft rejection but have failed to eliminate its progression entirely. Immune rejection due to species disparity is the major hurdle in the way of successful xenograft transplantation [1].

Hyperacute rejection (HAR), the initial barrier in pig-to-nonhuman primate organ transplantation, usually occurs within minutes to hours from the time of the transplant. It is mediated by pre-existing natural antibodies against the galactose-α-1,3-galactose (α-Gal) antigen and the activation of a complement cascade [2], [3]. Since Lai and colleagues first reported successful production of α-1,3-galactosyltransferase knockout (GT-KO) cloned pigs by somatic cell nuclear transfer (SCNT) [4], many research groups have developed α-Gal-deficient pigs [5], [6]. Recently, the development of biallelic GT-KO pigs has been reported using zinc finger nuclease (ZFNs) technology [7]. Transgenic mice expressing human α1,2-fucosyltransferase (HT), which competes with α1,3-galctosyltransferase for the same substrate *N*-acetyllactosamine, has shown to decrease α-Gal antigen expression by impeding the transfer of terminal galactose residue [8]. Numerous attempts at the depletion or inhibition of complement cascades have also been made to generate transgenic pigs that express the human C-reactive proteins (CRPs): decay-accelerating factor (hDAF, aka CD55) [9], membrane cofactor protein (hMCP, aka CD46) [10], and membrane inhibitor of reactive lysis (hCD59) [11]. These studies have provided evidence that organs from transgenic pigs expressing hCRPs are able to protect against complement-mediated cytolysis and exhibit prolonged xenograft survival [12], [13]. Moreover, the cells or organs from transgenic pigs expressing more than one hCRP as well as the combination of hCRP and HT have resulted in synergic protective effects against HAR [14], [15], [16]. Additionally, other approaches have indicated that cross-species incompatibilities in the coagulation system may be additional targets for genetic modifications in the second phase of rejection [17]. Considering the complexity of the human immune system, advanced strategies enabling multiple genetic modifications are therefore required to produce transgenic pigs in advance of clinical trials.

Many pigs with multiple transgenes have been obtained from consecutive stages of breeding with singly transgenic founders or screening after co-injecting separate vectors [18]. However, this process is not suitable for the examination of large animals because animal screening is expensive and time consuming.

Multiple transgenic approaches using polycistronic vector constructs, such as the internal ribosome entry site (IRES) and the self-cleavable 2A sequence, have been used widely as tools in basic research and in therapeutic applications [19], [20]. These approaches offer a highly efficient alternative by saving time and lowering the costs associated with producing multiple transgenic large animals. Combined with cloning technology, polycistronic vector constructs can provide a means to simplify founder reproduction throughout a number of consecutive generations [21].

In the present study, we tried to generate transgenic pigs that express three human transgenes, CD55, CD59, and HT, using both an IRES-mediated tricistronic vector system and SCNT to demonstrate the feasibility of this system as an efficient and reliable method for making multiple transgenic pigs. Our previous research and other studies have already determined the synergistic effects of the combined expression of CRPs and HT proteins as a strategy to improve long-term xenograft survival [22], [23]. Based on these studies, two human CRPs (hCD55 and hCD59) and H-transferase were selected. Our results showed higher transgene expression in transgenic pigs when compared with non-transgenic pigs. Moreover, most, but not all, endothelial cells from Tg piglets had protection from attack by the complement system, albeit at different efficiencies. We found that the expression of CRPs and the reduction of α-Gal in transgenic endothelial cells directly affect protection against complement-mediated cytolysis.

## Results

### Construction and Expression of Human CRPs (CD55 and CD59) and HT

**Figure 1:**
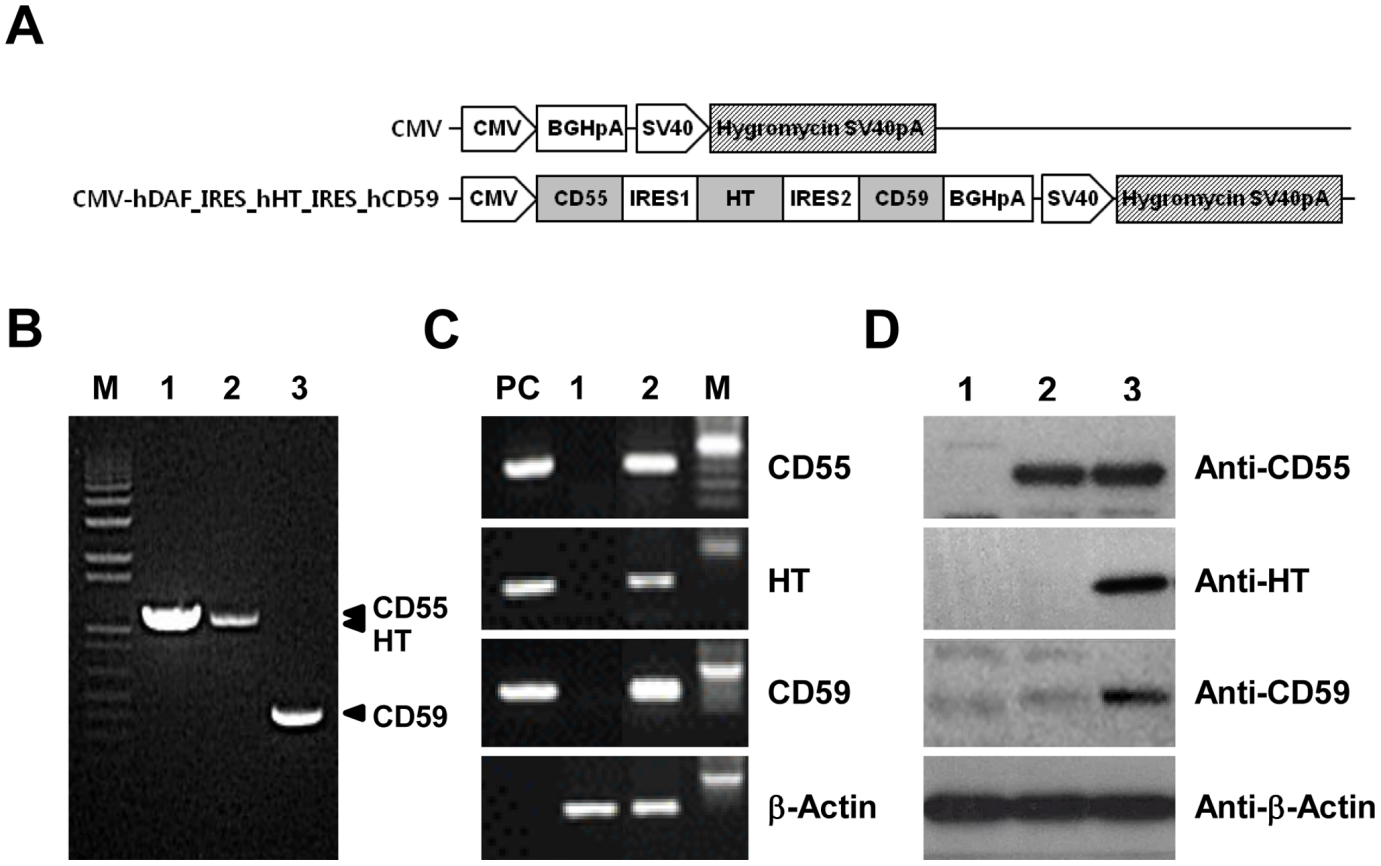
Construction of the tricistronic expression vector. (A) Schematic diagram of the IRES-mediated tricistronic vector encoding for human CRPs and H-transferase. Gene expression is driven by a pCMV promoter. (B) Cloning of hCD55, hCD59, and HT transgenes by RT-PCR. (C) Transcriptional assays were conducted by transient transfection of pig fetal fibroblast cells with triple vector. M, size marker; PC, tricistronic plasmid vector (positive control); 1, pcDNA3.1 transfectants; 2, pcDNA3.1-hCD55-IRES_HT-IRES_hCD59. (D) The original (lane 2) and the constructs with spacer sequence (lane 3) were tested by transient transfection of HEK293T cells. The expression of hCD55, HT and hCD59 proteins was analyzed by Western blotting. Total lysates (100 µg/lane) were loaded and were blotted with anti-CD55 (DAF), anti-CD59, anti-HT, and anti-β-Actin.

The first objective of this study was to generate a polycistronic vector to express multiple transgenes using encephalomyocarditis virus (EMCV) IRES. Figure 1A illustrates a schematic map of EMCV IRES-based tricistronic (triple) vectors that were designed to coexpress hCD55, hCD59, and HT under the control of a single porcine cytomegalovirus (pCMV) promoter. To evaluate the level of single-tricistronic mRNA, porcine fibroblasts was transiently transfected with the triple vector. The hCD55, hCD59, and HT genes were observed to be consistently coexpressed (Figure 1C). To evaluate whether the triple vector exerts a similar effect on the activity of the downstream genes, HEK293T cells were transiently transfected with the vector. Results showed that all the proteins were not consistently coexpressed in original tricistronic constructs (Figure 1D, lane 2). No detectable expression of the second cistron was observed and the expression of third cistron was much lower compared with the first one. To overcome this problems related to downstream attenuation with IRES, we inserted an unstructured spacer sequence (80 bp in length) to spatially separate the IRES and the cistrons. This separation can provide proper IRES folding without excessive structural interference or high expression levels of the cistron placed downstream of IRES [24]. We found that the expression of HT and hCD59 did not present the problems frequently encountered with IRES-mediated vectors, but HT and hCD59 appeared to have a relatively low level of expression compared with hCD55 (Figure 1D, Lane 3). Based on these findings, porcine fetal fibroblasts (pFF) of both sexes were transfected with the linearized triple vector and then were used as donor cells for SCNT.

### Generation of Transgenic Pigs Coexpressing Human CRPs (hCD55 and hCD59) and HT

The relative developmental competencies of embryos cloned with non-transgenic control (non-Tg) and triple-transgenic (Tg) male cells were evaluated. As shown in Table 1, the cleavage and blastocyst rates in the Tg cloned male (62.6% and 20.5%) and female (66.6% and 21.3%) embryos were noted to be similar to those of non-Tg clones (63.4% and 18.1%). Additionally, transgene expression in the male Tg cloned blastocysts was confirmed using RT-PCR analysis, whereas these gene expressions were not detected in non-Tg embryos (data not shown). To generate Tg cloned pigs, 100 reconstructed oocytes on average were surgically transferred into the oviducts of naturally cycling sows on the first day of standing estrus.

**Table 1:** The *in vitro* development of cloned embryos from normal and transgenic fetal fibroblasts.

Donor nuclei		No. examined	No (%).Cleaved	No (%). Blastocysts
Non Tg	(male)	298	189 (63.4±2.2)	54 (18.1±1.0)
Triple Tg	(male)	322	202 (62.6±1.5)	66 (20.5±0.6)
	(female)	245	163 (66.6±1.2)	52 (21.3±1.7)

The number of replicates was 6. The cleavage and blastocyst rates were counted on day 2 and at day 7, respectively. Data expressed show mean values ± SEM.

There was no significant difference in developmental rates among the groups.

**Figure 2:**
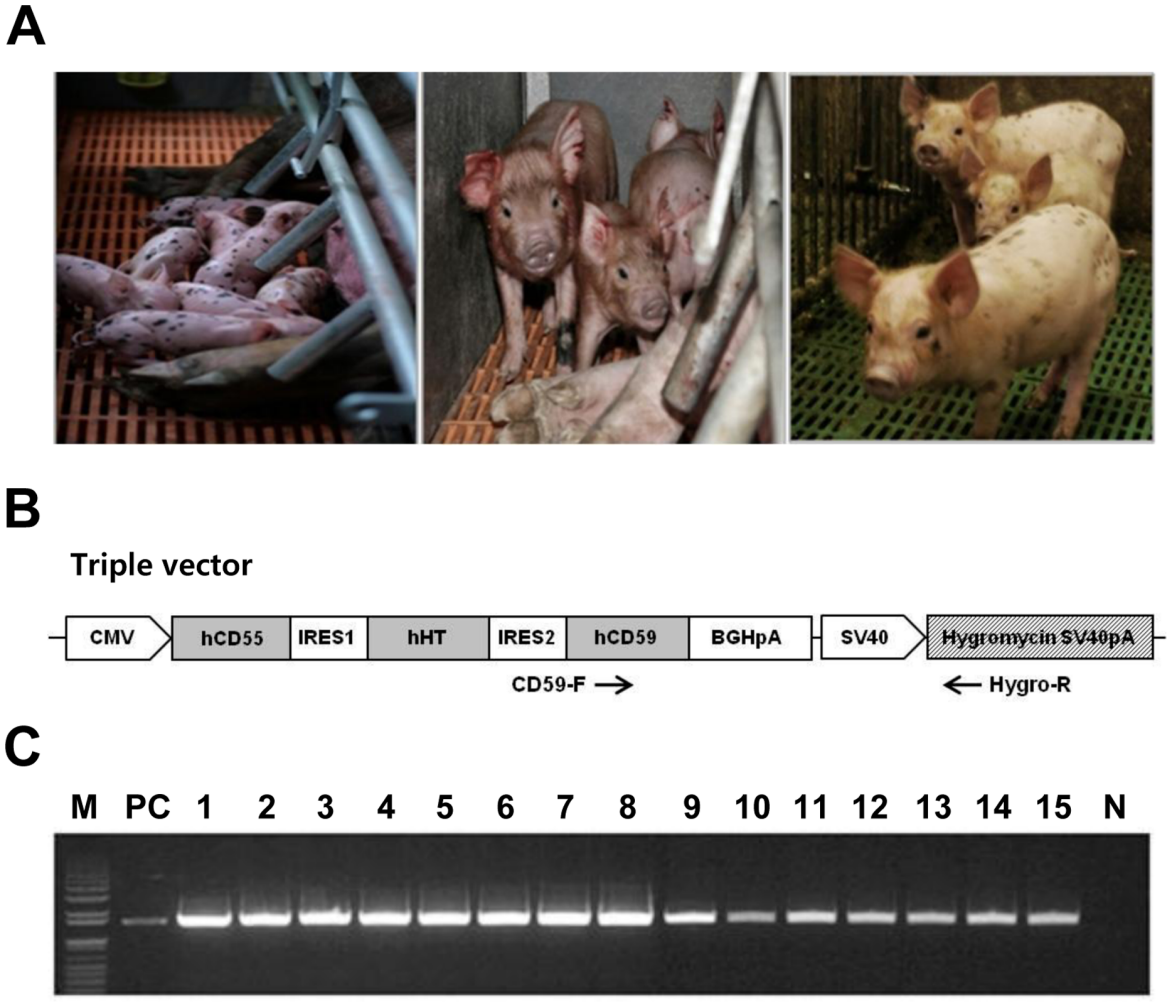
Production of transgenic pigs coexpressing hCD55, hCD59, and HT. (A) human CD55, CD59, and HT-transgenic piglets produced by SCNT. Left panel: female clones, middle and right panel: male reclones (B) Two different primer sets were used to genotype for the transgene. (C) M, size marker; PC, Triple plasmid vector as a positive control; 1–15 triple-transgenic piglets; N, non-transgenic pigs as a negative control.

**Figure 3:**
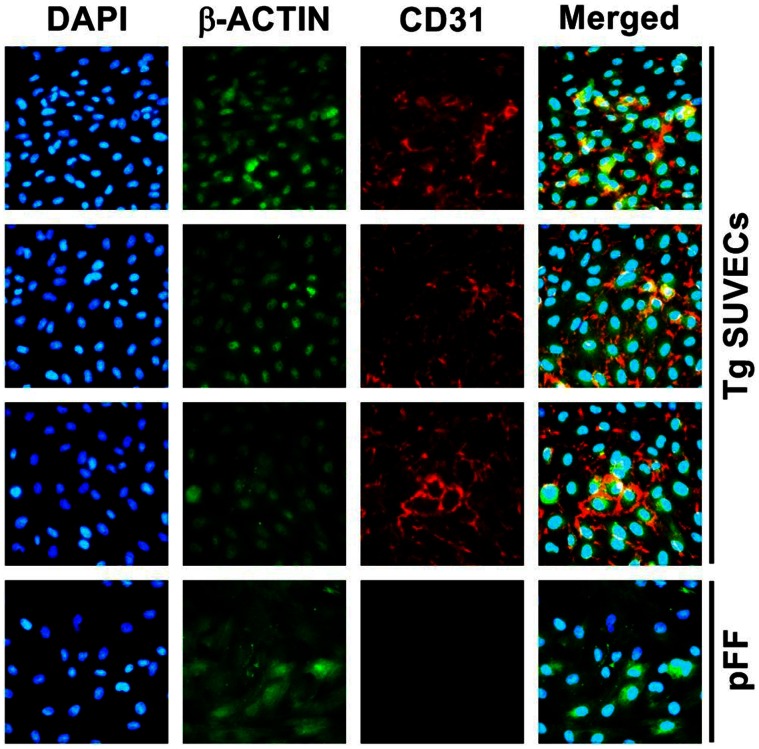
Primary Swine Umbilical Vein Endothelial Cells from triple-transgenic pigs. Primary cultured SUVECs were characterized by immunostaining with CD31 (red) and actin filaments (green), and the nuclei was counterstained with DAPI (blue).

**Figure 4:**
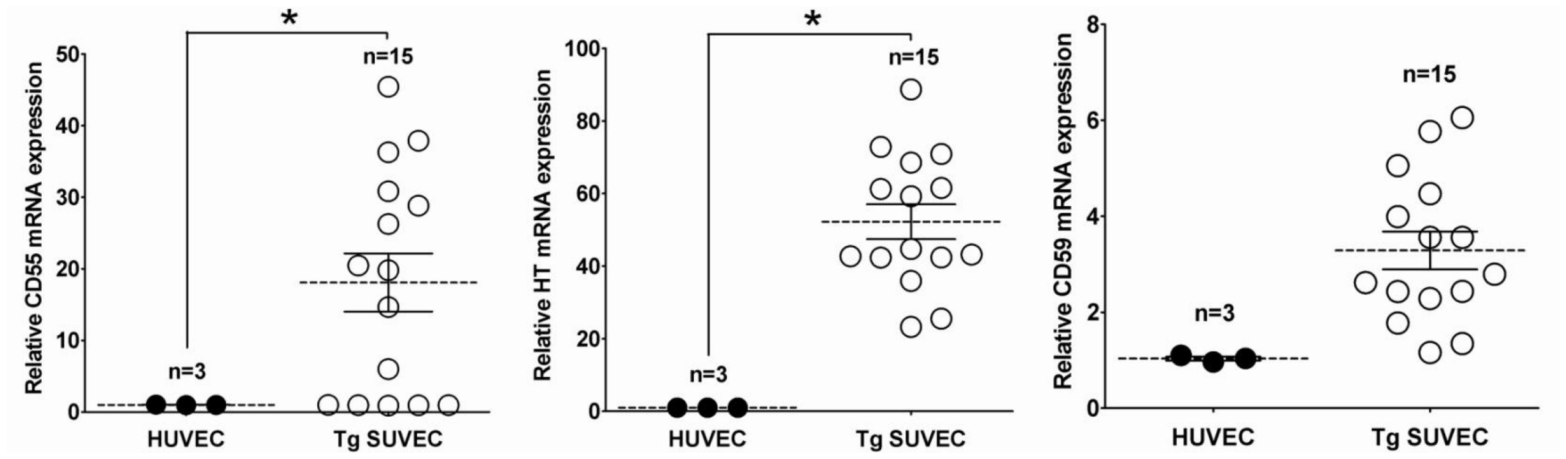
mRNA expression patterns of the transgenes in transgenic SUVECs. Scattered dot plots represent mRNA transcript levels for hCD55 (left), HT (middle), and hCD59 (right) in individual SUVECs. Each value derived from transcripts of the transgene in Tg SUVECs after normalization relative to β-Actin (internal control) genes, were compared with that of a HUVEC defined as 1.

The SCNT result of Tg male cell lines were stillborn or died shortly after birth (the pregnancy rate was 20%) and were therefore recloned using ear fibroblasts derived from the cloned piglet that had the highest expression of transgenes among the littermates (data not shown). The overall pregnancy rate was 41.6% for recloned males and 50% for females as a result of SCNT (*n* = 6). Sixteen live male and eighteen female Tg piglets from each of three gilts were born vaginally (Table 2 and Figure 2A). Porcine umbilical cords freshly obtained from the piglets were processed to confirm the integration of transgenes by PCR analysis (Figure 2B, C) and then used to isolate endothelial cells through collagenase digestion of the interior of the umbilical veins, as previously reported [25]. Primary cultured swine umbilical vein endothelial cells (SUVECs) were from Tg (four males and 11 females) and non-Tg piglets (three males) that were characterized by immunohistochemical (IHC) staining with anti-CD31 (PECAM-1) antibody, a common marker for endothelial cells (Figure 3). Fetal fibroblasts, used as a negative control cells, were found to be negative for this marker. We also analyzed transgene mRNA expression in SUVECs by qPCR to determine the performance of the tricistronic vector system. Human umbilical vein endothelial cell (HUVEC) and non-Tg SUVEC were used as the control in this study. In contrast to the high expression of these genes found in most Tg SUVECs rather than HUVEC, their transcriptional levels varied among the SUVECs (Figure 4). Of three transgenes, HT exhibited the highest expression pattern when compared with that of HUVECs. Low expression levels of hCD59 were found in several of the Tg SUVECs (#1, #7, #8, #10, and #15). The mean values of hCD55 and HT mRNA expression were significantly higher in Tg SUVECs than in HUVEC (*P*<0.05). The expression of hCD59 was not significantly higher and differed considerably between Tg SUVECs and HUVEC (*P* = 0.0564). A degree of expression heterogeneity among individual SUVECs was observed. However, the level of heterogeneity was similar among the transgenes indicating that variations between the transcript levels may be due to individuality among the clones.

**Table 2:** The *in vivo* development of triple cloned male and female embryos.

Cell line	No. experiments	No. embryos transferred	No.(%) pregnancy*	No. live offspring†
Male Tg	5	514	1 (20.0)	4
Male re Tg§	12	1378	5 (41.6)	16
female Tg	6	635	3 (50)	18

* An initial pregnancy diagnosis was examined via ultrasonography at 27 to 30 days after embryo transfer.

† all of the cloned piglets were vaginally delivered.

§ they were recloned using the ear fibroblasts derived from the cloned piglet that was seen in the highest expression of transgenes among the littermates.

### In vivo Transgene Expression in Various Organs of a Transgenic Piglet

**Figure 5:**
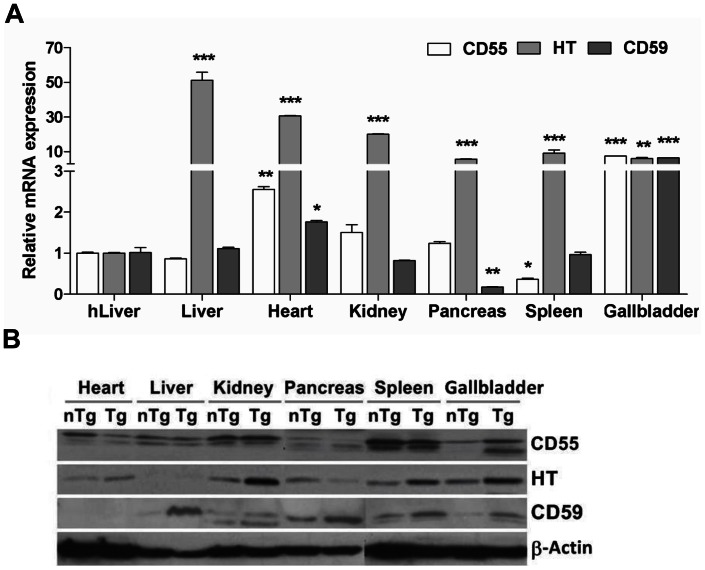
Transgene expression levels at different organs of a transgenic pig. (A) Each value derived from transcripts of the transgene in various organs of a transgenic pig (#1), after normalization relative to β-Actin (internal control) genes, were compared with that of a human liver sample defined as 1. (B) The expression of hCD55, hCD59, and HT proteins was analyzed by Western blotting. The results showed that the transgenes were detectable in most, but not all organs examined (heart, liver, kidney, pancreas, spleen, and GB).

**Figure 6:**
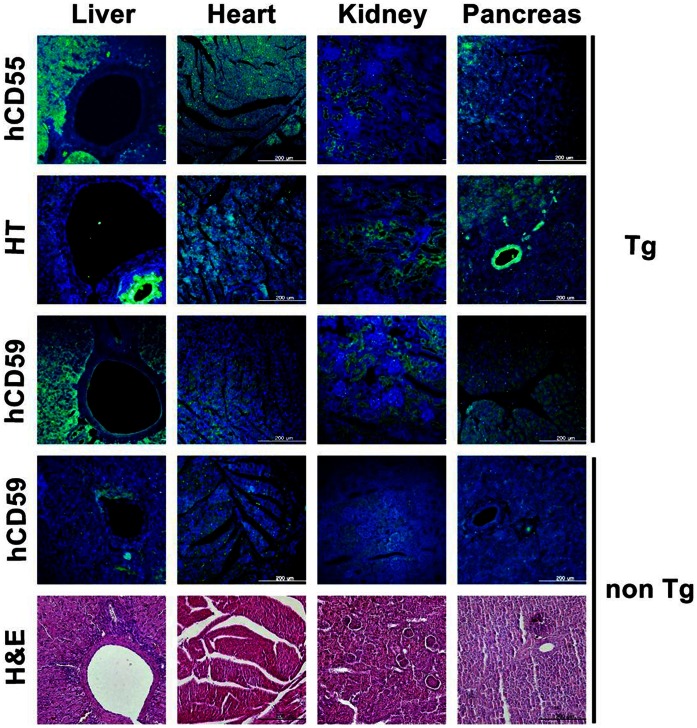
Immunohistochemical analysis in tissue sections of a transgenic piglet. Cryosections of the tissues were subjected to liver, heart, kidney and pancreas from a piglet. Frozen tissues were sectioned and immunostained with antibodies (green) as described under the Materials and Methods section. Nuclei were visualized by DAPI staining (blue). The images were taken using an inverted epifluorescence microscope. hCD55 and HT staining were barely detectable in tissue sections from non Tg, which were not included. Scale bar, 200 µm.

A randomly selected male Tg piglet (1 week old, Tg #1) was sacrificed, and then *in vivo* transgene expression levels were analyzed in various organs including the heart, liver, kidney, spleen, aorta, and inferior vena cava (IVC) by quantitative real-time PCR (qPCR) analysis, Western blotting, and IHC staining. The results revealed coexpressed transgene transcripts of hCD55, hCD59, and HT in most of the organs tested (Figure 5A). The expression level of HT was significantly greater in the Tg organs than that in the control human liver sample (*P*<0.0001). However, the other two CRP transgenes exhibited a significantly higher expression level, in the heart and IVC for hCD55 and in the heart for hCD59, when compared with the control (*P*<0.05). The hCD59 gene revealed a lower level of expression in the pancreas and aorta than in the control. As a control, we next analyzed protein expression levels in the various organs of Tg and non-Tg piglets by Western blot (Figure 5B) and IHC (Figure 6). In this analysis, we could not distinguish between human and pig proteins for these genes either by Western blotting or IHC, despite validation by most commercially available antibodies. This may mean that there is very little genetic difference between humans and pig species. As shown in Figure 5B, HT and hCD59 were observed at higher expression levels in Tg organs than those in non-Tg controls. However, there was no difference in the expression level of hCD55 between them. For HT, a strong positive staining in the intrahepatic ducts was noted on IHC (figure 6) but HT could not be detected in the liver tissue by Western blotting (indeed, the results from western blot and IHC were not consistent). hCD55 and hCD59 were expressed in a broad range of Tg tissues, but only very weakly expressed in non-tg tissues. Additionally, different expression intensities for antibodies were obtained from the organs. These results indicated that differences in expression levels among organs may be due to the organ specificity of the promoter or the protein synthesis activities of the different organs.

### Functional Analysis in SUVECs Isolated from Tricistronic Transgenic Pigs

**Figure 7:**
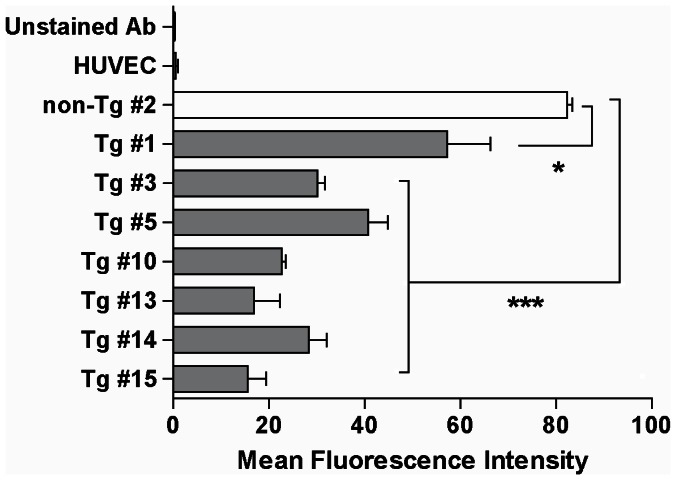
The expression of the α-Gal epitope in SUVECs by flow cytometry analysis. The bar graphs represents the average mean fluorescence intensity of Gal α-1,3-Gal antigen in Tg SUVECs (n = 7) compared with non-Tg SUVECs (n = 1) and HUVECs (n = 1). SUVECs were stained with GS-IB4 lectin, which binds to the Gal α-1,3-Gal antigen. The stained cells were analyzed with a FACS. The binding of GS-IB4 was decreased by 29.5% in Tg SUVECs compared with non-Tg SUVEC (P<0.0001). HUVEC was negative for GS-IB4 lectin staining. Data are expressed as the mean ± SDS in three independent experiments.

**Figure 8:**
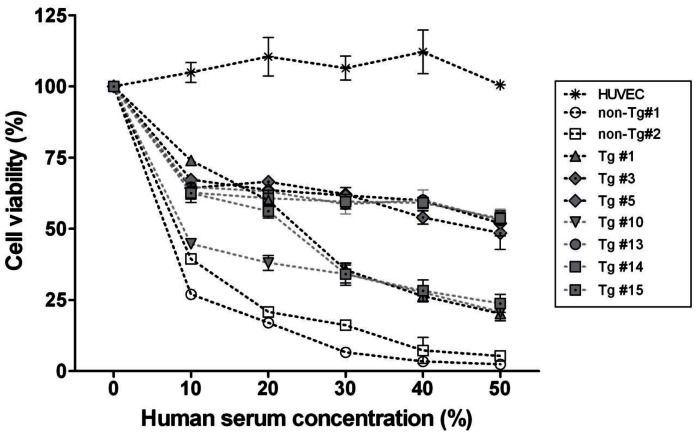
Human serum-mediated cytolysis of SUVECs. SUVECs from non-Tg (n = 2) and Tg (n = 7) were incubated for 6 h in MEM culture medium containing serial dilutions of normal human serum. Cytolysis rate was calculated based on the counts of live and dead cells. Mean ± SEM tested in three independent experiments are presented (P<0.001).

To verify the functional significance of Tg pig cells by the triple-vector system, all eight of the SUVECs (seven Tg and one non-Tg) were used. The cell-surface expression of α-Gal epitope in Tg and non-Tg SUVECs was measured by flow cytometry analysis with anti-GS-IB4 lectin (Figure 7). Our results showed that the α-Gal expression was down-regulated in all Tg cells compared with non-Tg. As shown in Figure 7, the average mean fluorescence intensity (MFI) of Tg was reduced by approximately 70.5% compared with that of non-Tg #2 SUVEC (Tg #1, *P*<0.05; Tg #3, #5, #10, #13, #14, and #15, *P*<0.0001). These results indicate that the cell-surface expression of α-Gal epitope was down-regulated by HT expression in Tg pig tissues. Based on this observation, we carried out an investigation of the protective effects of Tg SUVECs against cytolysis at various serum concentrations for 6 h. The results (Figure 8) showed that the survival rates of all Tg SUVECs were significantly higher than those of non-Tg SUVECs at all concentrations (e.g., at 50% serum concentration, the mean values were Tg #1, 20.2%; #3, 48.5%; #5, 51.9%; #10, 20.8%; #13, 52.9%; #14, 53.0%; and #15, 23.8%, and non-Tg #1, 2.4% and #2, 5.3%) (*P*<0.05). Increased serum concentration did not affect the overall survival rate in several Tg SUVECs (#3, #5, #13, and #14), whereas survival was gradually decreased with increased serum concentration in the other Tg (#1, #10, and #15) and all non-Tg SUVECs. The Tg SUVECs that displayed highly reduced α-Gal epitope expression had an increased survival rate at high levels of serum. Moreover, the cells with higher transgene expression levels (#5, #13, and #14) resulted in either down-regulation of α-Gal epitope expression or a greater level of protection against cytotoxicity. Conversely, the cells with lower transgene expression (#13 and #14) were less effective in attenuating α-Gal down-regulation. These results indicate that coexpression of hCD55, hCD59, and HT using the triple-vector system markedly enhanced the resistance of endothelial cells to human complement attack, and the increased viability of Tg SUVECs was accompanied by an increase in transgene expression.

## Discussion

Recently, pigs with genetically engineered organs have emerged as xenograft donors with large advantages, including anatomical and physiological similarities to humans. A number of transgenic pigs have been generated to prevent immunological rejection of transplanted organs. Several strategies have been attempted to reduce or eliminate the α-Gal epitope, which acts as a major trigger for HAR in xenotransplantation. The xenograft rejection mechanisms include several immunological processes that need to be considered through multiple genetic modifications of the donor pig in order to be successful in long-term xenograft survival [26]. Breeding processes with singly transgenic founders have generated many transgenic pigs with multiple modifications. Such strategies are time consuming and expensive, thereby requiring considerable effort. Multicistronic vectors have become effective tools for multigene transfer in biological research and gene therapy applications. Deng et al. [27] recently reported multi-transgenic pigs that coexpress the four fluorescent proteins via a single round of SCNT using a 2A peptide-based polycistronic vector system. The effectiveness of this multiple-gene-transfer system was clearly observed in cell-based experiments with pigs, but no such strategy has yet been applied in an in vivo context for xenotransplantation approaches [23], [28]. Therefore, inspired by the previous studies, we aimed to demonstrate the feasibility of this method as an efficient and reliable approach for generating multiple transgenic pigs for use in xenotransplantation. Herein, we describe the production of multiple transgenic pigs that express three human transgenes, hCD55, hCD59, and HT, through an IRES-mediated tricistronic vector system and SCNT.

As one of the strategies to overcome HAR, the coexpression of hCRPs and HT in organs from transgenic pigs has been shown to increase graft survival in primates [14], [29], [30]. It has been reported that when hCRPs are used in combination with HT, an additive protective effect against human serum mediated cytolysis results [31]. Similarly, in our previous study on the triple combination of hCRPs and HT, we showed that pig cells with these combined genes exhibited increased resistance against human complement-mediated cytolysis [23]. The present results showed that the expression of transgenes was significantly higher in Tg SUVEC cells compared with that in HUVEC. Flow cytometry analysis also showed that the Tg SUVEC had greatly reduced expression of the α-Gal epitope and tended to have relatively higher survival rates when compared with non-Tg SUVEC, as well as relatively high α-Gal expression values. This is consistent with previous studies on different cells from transgenic pigs, which have suggested that the expression level of HT is directly correlated with reduction in α-Gal expression, which in turn could affect protection against attack by the complement system [8], [31].

The present study showed that the levels of HT and CD59 were expressed at higher levels in the various Tg organs than in non-Tg organs. However, there was no difference in CD55 expression between Tg and non-Tg organs. Similarly, CD55 mRNA expression in five Tg SUVECs was much weaker than that in HUVEC. These Tg cells with weak CD55 expression exhibited a reduced protective effect against cytolysis. Although the reason for the very weak CD55 expression in several Tg SUVECs remains elusive, several putative causes have been proposed. For example, low or variable level of expression may be attributable to the insertion site of transgenes on the chromosome, resulting in a complex position effect. It may also be caused by a resistance mechanism of transgene-induced post-transcriptional gene silencing. Our data also indicated variable and spatially limited expression patterns of the transgenes in some organs. It has been shown that the inconsistency of transgene copy numbers gives rise to variable transgene expression [32]. Aside from the copy number of the transgene DNA, variability in transgene expression was found even in recloned piglets with the same genetic background. It was observed by IHC staining that HT in particular was expressed in oval and ductal cells in both the liver and the pancreas, in contrast to the undetectable levels found in these organs by Western blot. Therefore, it may be possible to explain these tissue-specific differences in translational sensitivity or in pCMV promoter preference among tissues [33], [34], [35]. Such variability of expression that is present in different organs and individuals could also lead to different responses to complement-mediated injury.

The construction of the polycistronic vector used in this study was achieved by EMCV-IRES, which has been proven to coexpress multiple transgenes in various cell types with high translation efficiency [36], [37]. By contrast, the translation of the cistrons located downstream of IRES have been shown to be greatly reduced and may even be absent [38]. Such downstream attenuation problems did not occur with the present vector system when an unstructured spacer element was introduced to help remove this limitation, at least to some extent. The spacer allows for proper IRES folding without excessive structural interference and mediates high expression levels of the second cistron [24]. Many reports have demonstrated that 2A peptides are more feasible in multiple-gene expression with a higher cleavage efficiency at the 2A site than are the corresponding IRES constructs [39], [40]. “Skipping” inefficiencies at the downstream 2A sites have been reported previously in mouse and pig studies. [27], [41]. These results suggest that although polycistronic vectors have proven to be effective tools for delivering multiple genes, the efficiency and effectiveness of these systems require further enhancement.

In this study, we demonstrated the generation of multiple transgenic pigs through a polycistronic vector system and SCNT for xenotransplantation research. Our results show that most, but not all, of the endothelial cells from Tg piglets demonstrated the protective effect of the complement system against attack, albeit with different efficiencies. Our results were also consistent with previous studies in showing that the expression of CRPs and the reduction of α-Gal in transgenic endothelial cells directly affects protection against complement-mediated cytolysis [31], [42], [43], [44]. Additionally, SUVEC provides a useful tissue model for performing functional analyses for xenograft organs with minimal sacrifice. These considerations led us to conclude that combining the two technologies offers the advantage of rapid generation of multiple transgenic pigs and is especially well suited for multiple genetic modifications of large animals with long gestation periods. This method may provide a useful means of further investigating the mechanisms of synergistic action among human CRPs and other proteins in response to acute-phase reactions so as to accelerate the production of transgenic animals for successful xenotransplantation and resolve the shortage of organs from human donors.

## Materials and Methods

### Ethics Statement

Normal human serum for complement cytolysis assays were prepared from blood donated by healthy volunteers after written informed consent and in accordance with the principles in ethical conduct as stated in the “National Statement on Ethical Conduct in Human Research”, documented by the Ministry of Education, Science and Technology (MEST) of the Republic of Korea. Sooam Biotech Research Foundation Institutional Review Board has reviewed and approved the study protocol of serum analysis and informed consent forms according to ethics requirements (SBRF-IRB-2010-1). The pig experiments were carried out in strict accordance with the recommendations in the Guide for the Care and Use of Laboratory Animals of the National Veterinary and Quarantine Service, and each study was approved by the animal ethics committee of Sooam Biotech Research Foundation (license number AEC-20081021-0001).

### Cloning and Tricistronic Vector Construction

The cDNAs of human CD55 (DAF), CD59, and HT were prepared by RT-PCR with the following oligonucleotides corresponding to the reported cDNA sequence of CD55 (GeneBank Accession No. DQ893935.2): 5′-ACA TGG TAC CTC CAC CAT GAC CGT CGC GCG-3′ (forward); 5′-TAG GAT CCC TAA GTC AGC AAG CCC AT-3′ (reverse), CD59 (GeneBank Accession No. BC001506.2): 5′-GCA ACC CGG GAT CAC AAT GGG AAT CCA AGG-3′ (forward); 5′-CGT CTA GAT TAG GGA TGA AGG CTC CAG GCT-3′ (reverse) and HT (GeneBank Accession No. NM_000148.3): 5′-CAG CTC GGC CAT GTG GCT CC-3′ (forward); 5′-TGG CTC TCA AGG CTT AGC CA-3′ (reverse), and using the total RNA of human whole blood (Figure 1B). The combination of cDNA and IRES were subcloned into a mammalian expression vector pcDNA3.1-Hygro between a cytomegalovirus CMV promoter and bovine growth hormone (BGH)-polyadenylated sequence (Invitrogen). The tricistronic expression vector for co-transcription of hDAF, hCD59, and HT genes was constructed using two IRES. All cDNAs were confirmed by DNA sequencing.

### Cell Culture and Transfection

Adult fibroblast cells were obtained from abdominal skin biopsy, and fetal fibroblast (pFF) cells were obtained from a day 27 pregnant Yucatan minipig that had mated naturally. The adult tissue samples were cut into small pieces (approx. 1 mm) with a scalpel. Dissected tissues were then cultured in Dulbecco’s modified Eagle’s medium (DMEM; Gibco-BRL) with 10% fetal bovine serum until confluent. Cells were frozen in DMEM with 10% fetal bovine serum (FBS) and 10% dimethyl sulfoxide. HEK293T cells, a cell line derived from human embryonic kidneys, were cultured in Dulbecco’s Modified Eagle’s Medium containing antibiotics and supplemented with 10% FBS at 37°C in 5% CO_2_.

Before transfection, cells were cultured at a density of 3×10^5^ cells/well in a 6-well plate for 24 h and then transfected with 2 µg of linearized DNA in NruI using Lipofectamine LTX™ Reagent (Invitrogen) as suggested by the manufacturer. At 24 h after transfection, the transfected cells were passaged and selected with 0.1 mg/ml of Hygromycin (Invitrogen) for 3–4 weeks. To confirm the stable integration of the tricistronic vector, the genomic DNA was extracted from the transfected clones and used as templates for PCR.

### Generation of Transgenic pigs

In vitro embryo production using SCNT was performed as previously described in our previous studies [45]. Porcine ovaries were provided by the regional slaughterhouse (Hyup-Shin, Anyang, Korea), collected from prepubescent gilts and transported to the laboratory within 1 h at 37°C. Briefly, enucleation was carried out in TL–HEPES supplemented with 0.4% bovine serum albumin (BSA) and 5 mg/ml cytochalasin B. In vitro matured denuded oocytes were enucleated by aspirating the polar body and MII chromosomes with an enucleation pipette (Humagen, Charlottesville, VA). After enucleation, a donor cell was introduced into the perivitelline space of an enucleated oocyte. Fusion of injected oocytes was induced in a fusion medium (280 mM mannitol, 0.001 mM CaCl_2_, and 0.05 mM MgCl_2_) by two DC pulses (1-s interval) of 2.0 kV/cm for 30 µs using a BTX-Cell Manipulator 200 (BTX). After fusion, oocytes were incubated for 1 h in TL–HEPES. The reconstructed oocytes were activated by an electric pulse (1.0 kV/cm for 60 µs) in an activation medium (280 mM mannitol, 0.01 mM CaCl_2_, 0.05 mM MgCl_2_), followed by 4 h of incubation in PZM3 medium containing 2 mmol/l 6-dimethylaminopurine. Embryo transfers were performed at a research farm (Department of Livestock Research, Gyeonggido Veterinary Service, Korea). Approximately 100 reconstructed oocytes were surgically transferred into the oviducts of naturally cycling gilts (approx. 9 months old) on the first day of standing estrus. Pregnancies were confirmed by ultrasound on day 30 (day 0 was the day of SCNT).

### Isolation of Swine Umbilical Vein Endothelial Cells (SUVEC)

Swine umbilical vein endothelial cells (SUVEC) were obtained from freshly harvested porcine umbilical cord veins by treatment with 0.25% trypsin, as previously described by Baudin et al. [25]. The cells were washed with phosphate-buffered saline (PBS), and all effluent was collected in the conical tube containing the FBS. For expansion, isolated SUVEC were cultured in the endothelial growth medium (medium 199 containing 20% FBS, antibiotics, and supplemented 30 ng/ml vascular endothelial growth factor (VEGF, Sigma), 10 ng/ml EGF (Invitrogen), 10 ng/ml bFGF (Invitrogen), and β-ME (Gibco-BRL)). SUVEC were grown to confluence in a fibronectin-coated tissue culture dish and used for experiments between passages 3 and 6.

### Isolation of Total RNA

Total RNA was isolated from transient transfected porcine cells with triple vectors using the RNeasy mini kit (QIAGEN) at 48 h post-transfection. Messenger RNA from the individual blastocysts was extracted using the Dynabeads mRNA Direct Kit (DynalAsa, Oslo, Norway) following manufacturer’s instructions. Various tissues were obtained from a 3-day-old female transgenic pig. Then, total RNA was isolated using TRizol reagent™ (Invitrogen) for quantitative real-time PCR to determine the expression of hCD55, hCD59, and HT mRNA in various porcine tissues, ear fibroblast cells, and umbilical vein endothelial cells.

### Quantitative Real-time PCR

Reverse transcription was performed with 2 µg of total RNA, random primer, and M-MLV RT (Invitrogen) according to the manufacturer’s instructions. Quantitative real-time PCR was performed using SYBR Master Mixes (Applied Biosystems) with ABI PRISM 7300 Sequence Detection system (Applied Biosystems). Real-time PCR was performed with specific primers to detect the expression of human CD55 using 5′-CCA GGA CAA CCA AGC ATT TT-3′ as the forward primer and 5′-TGG TTA CTA GCG TCC CAA GC-3′ as the reverse primer. Human CD59 was detected using 5′-GTC ACA ACC CGC TTG AGG-3′ as the forward primer and 5′-GGA TGT CCC ACC ATT TTC AAG-3′ as the reverse primer; and human HT was detected using 5′-AGC CAT CGT CAG CTC TGC-3′ as the forward primer and 5′-GGC CAC TGG GGG TGT CAC-3′ as the reverse primer. Primary cultured HUVEC cells were purchased from Innopharmascreen Inc. (Asan, Korea) and were used as a positive control. SUVECs obtained from a non-Tg cloned pig were used as a negative control for hCD55, hCD59, and HT expression.

### Western Blot Analysis

Lysates from whole cells were resolved in sodium dodecyl sulphate (SDS) sampling buffer. Following SDS-polyacrylamide gel electrophoresis, proteins were transferred to nitrocellulose membranes. The membranes were blocked with 5% skim milk and 0.05% Tween 20 in PBS overnight at 4°C and then blotted with rabbit anti-CD55 (1∶1,000; Sigma), rabbit anti-HT (1∶1000, LifeSpan Biosciences Inc), goat polyclonal Ab N-20 (1∶1,000; Santa Cruz), and mouse anti-β-Actin (1∶1,000; Santa Cruz) for 4 h at room temperature. The membranes were washed three times in 0.05% Tween 20 in PBS for 45 min and incubated for 1 h with secondary antibodies. After washing with 0.05% Tween 20 in PBS, the membrane was subjected to enhanced chemiluminescence detection analysis (GE Healthcare) using horseradish peroxidase-conjugated secondary antibodies (Chemicon).

### Immunohistochemical (IHC) Staining

Biopsies of cloned transgenic pig liver, heart, kidney, eye, aorta, IVC, and pancreas were formalin fixed, quickly frozen in liquid nitrogen, and then stored at −80°C in preparation for IHC studies. Frozen tissues were sectioned and stained with hematoxyllin and eosin and evaluated under light microscopy. Frozen sectioned tissue were permeabilized with 0.1% Triton X-100 and blocked with 1% BSA prior to staining with FITC-conjugated mouse anti-human CD55 antibody (1∶100, Biolegend), FITC-conjugated lectin from Ulex europaeus (1∶100, UEA-I, Sigma), and FITC-conjugated mouse anti-human CD59 antibody (1∶100, Biolegend). Samples were counterstained using with Prolong gold with DAPI (Invitrogen Cat # P-36931) prior to imaging. Images were acquired using inverted microscope with epiflorescent (Nikon TE2000; Nikon Corp., Tokyo, Japan). To confirm endothelial cells, cultured SUVECs were immunostained with purified mouse anti-pig CD31 (PECAM-1, Serotec) as described above. Isolated SUVECs were confirmed to be >90% by immunostaining with anti-CD31.

### Cell Staining for Identification of α-Gal Antigen

The SUVECs were diluted to 1×10^5^ cells per tube in FACS buffer. Surface expression of α-Gal antigens was measured by direct immunofluorescence using FITC-conjugated isolectin GS-IB4 (Sigma). Cells were incubated for 30 min at 4°C. Unstained cells were used as negative controls.

### Flow Cytometry Analysis

Cultured swine umbilical vein endothelial cells obtained from the transgenic piglet’s umbilical cord vein were examined for evaluation of the expression level of α-Gal antigen using FITC-conjugated lectin GS-IB4 (Sigma).

### Human Serum-mediated Cytolysis Assay

The viability of SUVECs obtained from the transgenic piglet’s umbilical cord vein after human serum treatment was measured by the WST-1 (Roche) cell proliferation assay system (Roche) using the manufacturer’s protocol. Normal human serum (NHS) to be used as a complement source was obtained from the blood of healthy volunteers in the laboratory and stored at −80°C until use. SUVECs from non-Tg and Tg pigs were plated on 96-well plates at 3×10^4^ cells/well in 100 µl M199 medium containing 10% FBS for 24 h prior to the assay and then incubated with increasing concentrations of normal human serum for 6 h at 37°C. Cell viability was determined based on the cleavage of the tetrazolium salt WST-1 (Roche) by mitochondrial dehydrogenases in viable cells relative to the no-serum-added condition using colormetric detection of the optical absorbance at 460 nm by a scanning multiwall spectrophotometer (Sunrise).

### Statistical Analysis

The data obtained in this study were analyzed using the GraphPad Prism statistical program (GraphPad Software, San Diego, CA). Data on flow cytometry analysis were examined using analysis of variance (ANOVA) followed by Dunnett’s test. Relative transcription levels between HUVEC and Tg SUVEC were analyzed using unpaired Student’s *t*-tests. All data are presented as mean values ± SEM. A probability of *P*<0.05 was considered statistically significant in all tests.
